# Smoking cessation, weight change, and incidence and progression of frailty: A longitudinal analysis of the US Health and Retirement Study

**DOI:** 10.18332/tid/231379

**Published:** 2026-09-17

**Authors:** Yixiao Liang, Ao Li, Lingtong Ren, Hu Ai

**Affiliations:** 1 Department of Cardiology, Beijing Hospital, National Center for Gerontology, National Clinical Research Center for Gerontology, The Key Laboratory of Geriatrics of NHC, Institute of Geriatric Medicine, Chinese Academy of Medical Sciences, Beijing, China; 2 Department of Respiratory and Critical Care Medicine, Beijing Hospital, National Center for Gerontology, National Clinical Research Center for Gerontology, The Key Laboratory of Geriatrics of NHC, Institute of Geriatric Medicine, Chinese Academy of Medical Sciences, Beijing, China

**Keywords:** smoking cessation, weight change, frailty, aging, Health and Retirement Study

## Abstract

**INTRODUCTION:**

Smoking cessation is important for healthy ageing, but accompanying weight change may influence subsequent functional vulnerability. We examined whether attained cessation duration and weight change across the observed cessation interval distinguished later frailty risk and frailty-index progression.

**METHODS:**

This longitudinal secondary analysis included 27020 Health and Retirement Study participants with self-reported smoking and body-weight data, and valid consecutive frailty assessments who were non-frail at risk-set entry during 1998–2020. Smoking status, body weight, and covariates were assessed biennially by structured interviews. Time-varying Cox models estimated incident frailty (frailty index ≥0.25), mixed-effects models assessed annual frailty-index change, and restricted cubic splines evaluated continuous associations.

**RESULTS:**

Over a median follow-up of 8.1 years, 10641 incident frailty cases occurred during 250439.9 person-years. Compared with current smokers, quitters before baseline had lower frailty risk (hazard ratio, HR=0.79; 95% CI: 0.75–0.84), whereas follow-up quitters overall did not (HR=0.98; 95% CI: 0.90–1.07). In the duration spline, HRs at 0, 7, and 10 years of attained cessation duration were 1.14 (95% CI: 1.01–1.28), 0.79 (95% CI: 0.67–0.93), and 0.73 (95% CI: 0.60–0.89), respectively. Among follow-up quitters, >10.0 kg versus 0.1–5.0 kg weight gain was associated with higher risk (HR=1.94; 95% CI: 1.44–2.61); standardized 2-year risks among eligible analytic intervals were 15.9% and 9.5%, respectively. The direct progression contrast was imprecise in Model 3 and was estimated at β=0.415 (95% CI: 0.040–0.790; p=0.030) after additional adjustment for interval-start frailty index.

**CONCLUSIONS:**

Longer cessation duration was associated with lower frailty risk. Substantial weight gain across the observed cessation interval was associated with higher absolute frailty risk among follow-up quitters. Smoking cessation remains the priority, but marked gain may help identify quitters who warrant continued weight and functional monitoring.

## INTRODUCTION

With the rapid aging of the population, frailty has become an increasingly important geriatric syndrome and a major health burden^1,2^. Frailty represents a multidimensional state of declining adaptive capacity, in which age-related deficits across biological, functional, and psychosocial domains reduce an individual’s ability to maintain health in response to stressors^2,3^. The frailty index, a widely used instrument developed by Rockwood et al.^4^ to measure frailty, provides a practical way to operationalize this construct by summarizing the proportion of age-related deficits present in an individual^4,5^. A higher frailty index has been consistently associated with adverse health outcomes, including falls, disability, hospitalization, cardiovascular disease, cognitive decline, and mortality^5,6^. However, frailty is not a static or inevitably progressive condition. Evidence from longitudinal studies suggests that individuals may transition between frailty states over time, and that improvement or stabilization of frailty status is possible^7^. Therefore, identifying modifiable exposures that influence frailty development and progression is important for informing prevention strategies before health deficits become firmly established.

Smoking is a plausible behavioral factor in this process. Its established effects on cancer, cardiovascular disease, chronic lung disease, physical function, and multimorbidity overlap with pathways involved in frailty development. Longitudinal studies have reported that current smokers are more likely to develop frailty, and systematic evidence suggests that smoking may predict worsening frailty status in community-dwelling older adults^8-11^. These findings point to smoking cessation as a potentially relevant strategy for frailty prevention^12^.

Smoking cessation reduces the risk of major chronic diseases and premature death^13,14^. Despite these benefits, smoking cessation is commonly followed by weight gain^15,16^. Concerns about post-cessation weight gain may discourage quit attempts and contribute to relapse^17^. Studies in North America have reported that post-cessation weight gain is generally modest but variable, with many quitters gaining <4.5 kg and a smaller subgroup experiencing substantial gain of ≥11 kg; much of this weight gain tends to occur during the early months after quitting and may persist over time^15,16^. More broadly, prior studies of adulthood weight change have linked both weight loss and substantial weight gain to higher frailty risk, suggesting that body weight change itself may be relevant to frailty development^18^. Because substantial weight gain may indicate greater adiposity and related metabolic or functional burden, weight gain after cessation could plausibly influence the health benefits of quitting, particularly for aging-related outcomes such as frailty^19-22^.

Evidence from cardiometabolic and mortality outcomes suggests that post-cessation weight gain may not uniformly attenuate the benefits of quitting^23,24^. In three large US cohorts, recent quitters had a higher short-term risk of type 2 diabetes, particularly with greater weight gain, whereas lower cardiovascular and all-cause mortality risks persisted despite weight gain^24^. Whether a similar balance applies to frailty remains unclear, and direct frailty evidence is limited. A recent study using the English Longitudinal Study of Ageing examined 2600 former smokers and found that longer time since smoking cessation was associated with lower frailty-index trajectories over 16 years; however, it classified former smokers by cessation duration at baseline and did not assess body weight change after quitting^12^. Thus, whether weight change across an observed cessation transition differentiates subsequent frailty risk among people who quit during follow-up remains unknown.

To address this gap, we used repeated biennial assessments from the US Health and Retirement Study to examine whether weight change across an observed cessation transition differentiates frailty risk among adults who quit smoking during follow-up. Rather than asking only whether former smokers differ from current smokers, we focused on whether this cessation-interval marker identifies subgroups of quitters with lower or higher subsequent frailty risk. We also examined annual frailty-index change to assess whether these patterns were reflected in deficit accumulation over time.

## METHODS

### Study population and design

This longitudinal secondary analysis used data from the Health and Retirement Study (HRS), a nationally representative cohort of US adults aged ≥50 years^25,26^. The HRS follows participants with core interviews conducted every two years, collecting detailed information on sociodemographic characteristics, health behaviors, medical history, functional status, and psychosocial factors. The present analysis used repeated assessments from 1998 to 2020, when the variables required to construct the frailty index (FI) and characterize smoking transitions were available in a comparable manner.

We constructed a time-varying person-period dataset in which each observation represented the interval between two consecutive HRS interviews. Smoking status, body weight, frailty index, and covariates were assessed at the start of each interval, and incident frailty was assessed at the next interview. Participants could contribute multiple risk intervals until incident frailty, death, loss to follow-up, or the end of follow-up, whichever occurred first. For the incident frailty analysis, participants entered the risk set at the first eligible interval in which they were non-frail at the interval-start assessment. Each eligible interval required valid smoking status, valid frailty assessment at the interval start and next interview, and follow-up time. The final incident frailty analytic sample included 27020 participants. The inclusion flow chart is shown in Supplementary file eFigure 1.

### Smoking status and weight change across the observed cessation interval

Smoking status was assessed at each interview using self-reported current and lifetime smoking information. Participants were classified as current smokers, past smokers, or never smokers at each interval start. To examine the timing of cessation, past-smoker person-time was further classified as quitting before baseline or quitting during follow-up. Quitters before baseline were participants who were not current smokers at their first eligible risk-set entry but reported a history of smoking. Past-smoker intervals that were not preceded by an observed current-to-noncurrent smoking transition within the analytic person-period data were also classified as quitters before baseline, whereas intervals after an observed transition from current smoking at the previous wave to non-current smoking at the current wave were classified as follow-up quitter person-time. For follow-up quitters, attained cessation duration was set to 0 at the first observed non-smoking wave after a current-smoking wave and increased by observed follow-up time during continued non-smoking within that episode.

Among follow-up quitters, weight change across the observed cessation interval was body weight at the first wave reporting non-smoking minus body weight at the preceding current-smoking wave. Because exact quit dates were unavailable, this approximately 2-year measure may capture weight change before and after cessation. It was categorized as: ≤0, 0.1–5.0, 5.1–10.0, and >10.0 kg^15,16^. These categories were used to distinguish no weight gain, modest weight gain, moderate weight gain, and substantial weight gain across the observed cessation interval. The category marked the observed cessation episode and was carried forward through continued non-smoking intervals; subsequent weight measurements did not reclassify the episode. Intervals after relapse were reclassified as current-smoking person-time. Among follow-up quitters, participants without valid body-weight measurements at either transition wave were excluded from analyses that classified quitters by cessation-interval weight change.

### Frailty assessment

Frailty was assessed at each wave using a 32-item frailty index based on the deficit accumulation approach^4,5,27^. The index included health deficits spanning chronic conditions, physical functioning and disability, sensory impairment, cognitive function, and psychological health^28-30^. It was calculated as the proportion of observed deficits present, with values ranging from 0 to 1; higher values indicated greater deficit accumulation. A valid frailty index required at least 26 of 32 items to be observed. Smoking status, body weight, body mass index, obesity, and weight loss were not included as frailty index items. Detailed items are shown in Supplementary file Table S1.

Incident frailty was defined as the first transition from frailty index <0.25 at the interval-start assessment to frailty index ≥0.25 at the next interview. For frailty progression, interval-specific annual frailty index change was calculated as the next-wave frailty index minus the interval-start frailty index divided by years of follow-up, and was expressed as percentage points per year.

### Covariates

Covariates were selected as potential confounders based on prior literature and availability in the HRS. Age (years), sex (male, female), race/ethnicity (White, Black, or Other), education level (lower than high school, General Educational Development, high school graduate, some college, or college and higher), marital status (married/partnered or not), and annual household income ($) (<20000, 20000 to <40000, 40000 to <80000, or ≥80000) were obtained by structured interview. Behavioral covariates were alcohol intake (non-drinker, ≤1, 1–2, or >2 drinks/day) and vigorous physical activity (<3 or ≥3 times/week). Body mass index (BMI) was calculated as weight (kg) divided by the square of the height (m), and categorized as: <25.0, 25.0–29.9, or ≥30.0 kg/m² for descriptive and subgroup analyses. Missing categorical covariate values were retained as missing/unknown (Supplementary file eTable 2).

### Statistical analysis

Baseline characteristics were described at the first interval-start wave when each participant entered the incident frailty risk set. Continuous variables were summarized as means and standard deviations, and categorical variables as frequencies and percentages. Overall p-values were calculated using one-way analysis of variance or Pearson chi-squared tests, as appropriate.

We used Cox proportional hazards models to estimate hazard ratios and 95% confidence intervals for incident frailty according to smoking cessation status and weight change across the observed cessation interval. Smoking status, the cessation-interval weight-change category, and covariates were updated at the start of each interview interval. Participants contributed person-time from their first eligible non-frail interval until incident frailty, death, loss to follow-up, or the end of follow-up, whichever occurred first. Separate models were fitted for the prespecified exposure definitions: overall smoking status, timing of smoking cessation, smoking cessation with cessation-interval weight change, and cessation-interval weight-change categories among follow-up quitters. Model 1 was stratified by 5-year age group and study wave. Model 2 further adjusted for sex, race/ethnicity, education level, marital status, and household income. Model 3 further adjusted for alcohol intake and vigorous physical activity.

Linear mixed-effects models were used to compare interval-specific annual frailty-index change across exposure groups. These models adjusted for 5-year age group, sex, race/ethnicity, education level, marital status, household income, alcohol intake, vigorous physical activity, and interval-start wave, with a random intercept to account for repeated observations within individuals. Current smokers were the reference group in the primary progression models. Among follow-up quitters, we additionally used 0.1–5.0 kg gain as the reference and compared Model 3 with a model further adjusted for interval-start frailty index. Coefficients were expressed as differences in annual frailty-index change, in percentage points per year; negative coefficients indicated slower accumulation relative to the corresponding reference group.

We used restricted cubic-spline Cox models to examine continuous associations of attained cessation duration and cessation-interval weight change with incident frailty. For attained cessation duration, current smoker person-time was the common reference in an unconstrained four-knot spline model, with the HR at 0 years estimated from the data. Estimates at each attained duration compare current smoker risk-set person-time with follow-up quitters who remained abstinent, alive, observed, and frailty-free to that duration. Separate indicators represented quitters before baseline and never smokers, and the never smoker estimate from the same model was displayed as a comparator. Weight-change analyses were restricted to follow-up quitters with valid weight data. Four knots were placed at the 5th, 35th, 65th, and 95th percentiles, with 0 kg as the reference; estimates were displayed from the 1st to the 99th percentile to limit tail extrapolation. Overall and nonlinear associations were evaluated using Wald tests. Models used the same covariate adjustment as Model 3. Prespecified subgroup analyses were performed by age, sex, body mass index, and vigorous physical activity. Interaction was assessed by comparing models with and without exposure-by-subgroup interaction terms.

To complement relative-effect estimates, we estimated model-standardized 2-year absolute risks among follow-up quitters using a pooled complementary log-log model with log (interval length/2 years) as an offset and participant-clustered robust standard errors. The model included the same covariates as Model 3, and risks were standardized to the covariate distribution of eligible follow-up-quitter person-intervals in the analytic sample. We report standardized risks, risk differences, risk ratios, and excess events per 1000 two-year person-intervals, using 0.1–5.0 kg gain as the reference. Standardization targeted eligible analytic follow-up-quitter person-intervals; observation-probability weighting for nonresponse or loss to follow-up was not applied.

Several sensitivity analyses were conducted to assess the robustness of the main findings, including use of an alternative frailty threshold of frailty index ≥0.20, exclusion of incident frailty events occurring within the first 2 years, repeating the primary Cox analysis after excluding participants with major chronic diseases at baseline to reduce potential reverse causation, Fine–Gray competing-risk models, and multiple imputation by chained equations for missing covariates. For frailty-index progression, an additional sensitivity analysis included participants with baseline frailty. Two additional complete-case sensitivity analyses among follow-up quitters addressed potential residual confounding. The first added BMI and frailty index measured at the wave preceding the observed cessation transition; the second added pre-cessation smoking duration, defined as age at that preceding smoking wave minus self-reported age at smoking initiation. In each sensitivity, Model 3 and the additionally adjusted model were estimated in the same complete-case sample with 0.1–5.0 kg gain as the reference. Fine–Gray competing-risk models treated death before incident frailty as a competing event and estimated sub-distribution hazard ratios for incident frailty. The proportional hazards assumption was assessed using Schoenfeld residuals for the primary Cox models, and no material violation was observed for the primary exposure contrasts. Two-sided p<0.05 indicated statistical significance. All analyses were performed using R version 4.4.1 (R Foundation for Statistical Computing, Vienna, Austria).

## RESULTS

### Baseline characteristics

A total of 27020 participants entered the time-varying Cox risk-set sample. At first entry, 4966 were current smokers, 10027 past smokers, and 12027 never smokers. Current smokers were younger than never smokers (mean 56.6 vs 59.4 years), had an education level of lower college (12.1% vs 28.7%), and were less often vigorously active ≥3 times/week (37.0% vs 43.3%); overall smoking-group comparisons were statistically significant for all Table 1 characteristics (all p<0.001). Current smokers also had a higher prevalence of chronic lung disease, whereas past smokers had higher baseline prevalences of diabetes, heart disease, stroke, and cancer.

**Table 1:** Participant characteristics at first entry into the time-varying Cox risk set, Health and Retirement Study cohort, USA, 1998–2020 (N=27020)

*Characteristics*	*Overall* *(N=27020)* *n (%)*	*Current smokers* *(N=4966)* *n (%)*	*Past smokers* *(N=10027)* *n (%)*	*Never smokers* *(N=12027)* *n (%)*	*p*
**Age** (years), mean (SD)	59.6 (10.1)	56.6 (8.3)	61.3 (10.1)	59.4 (10.4)	<0.001
**Sex**					<0.001
Male	12115 (44.8)	2425 (48.8)	5547 (55.3)	4143 (34.4)	
Female	14905 (55.2)	2541 (51.2)	4480 (44.7)	7884 (65.6)	
**Race/ethnicity**					<0.001
White	18130 (67.1)	3151 (63.5)	7220 (72.0)	7759 (64.5)	
Black	4554 (16.9)	1121 (22.6)	1391 (13.9)	2042 (17.0)	
Other	4318 (16.0)	691 (13.9)	1408 (14.0)	2219 (18.5)	
**Education level**					<0.001
Lower than high school	4902 (18.1)	1156 (23.3)	1783 (17.8)	1963 (16.3)	
GED	1225 (4.5)	381 (7.7)	495 (4.9)	349 (2.9)	
High school graduate	7769 (28.8)	1511 (30.4)	2820 (28.1)	3438 (28.6)	
Some college	6671 (24.7)	1315 (26.5)	2534 (25.3)	2822 (23.5)	
College and higher	6450 (23.9)	601 (12.1)	2394 (23.9)	3455 (28.7)	
**Marital status**					<0.001
Married/partnered	19947 (73.8)	3356 (67.6)	7649 (76.3)	8942 (74.3)	
Not married/partnered	7053 (26.1)	1606 (32.3)	2370 (23.6)	3077 (25.6)	
**Household income** ($)					<0.001
<20000	5703 (21.1)	1318 (26.5)	1902 (19.0)	2483 (20.6)	
20000 to <40000	6261 (23.2)	1260 (25.4)	2405 (24.0)	2596 (21.6)	
40000 to <80000	7451 (27.6)	1378 (27.7)	2890 (28.8)	3183 (26.5)	
≥80000	7605 (28.1)	1010 (20.3)	2830 (28.2)	3765 (31.3)	
**Body weight** (kg), mean (SD)	79.4 (17.9)	77.0 (17.3)	81.8 (17.6)	78.4 (18.3)	<0.001
**BMI** (kg/m²)					<0.001
<25.0	8670 (32.1)	2001 (40.3)	2867 (28.6)	3802 (31.6)	
25.0–29.9	10617 (39.3)	1854 (37.3)	4234 (42.2)	4529 (37.7)	
≥30.0	7225 (26.7)	1050 (21.1)	2774 (27.7)	3401 (28.3)	
**Frailty index,** mean (SD)	0.1 (0.1)	0.1 (0.1)	0.1 (0.1)	0.1 (0.1)	<0.001
**Vigorous physical activity** (times/week)					<0.001
<3	15314 (56.7)	3121 (62.8)	5381 (53.7)	6812 (56.6)	
≥3	11681 (43.2)	1838 (37.0)	4640 (46.3)	5203 (43.3)	
**Alcohol intake** (drinks/day)					<0.001
Non-drinker	16122 (59.7)	2527 (50.9)	5442 (54.3)	8153 (67.8)	
<1	7537 (27.9)	1313 (26.4)	3160 (31.5)	3064 (25.5)	
1–2	2009 (7.4)	567 (11.4)	909 (9.1)	533 (4.4)	
>2	1258 (4.7)	519 (10.5)	491 (4.9)	248 (2.1)	
**Diabetes**	2715 (10.0)	393 (7.9)	1155 (11.5)	1167 (9.7)	<0.001
**Heart disease**	2718 (10.1)	383 (7.7)	1311 (13.1)	1024 (8.5)	<0.001
**Stroke**	658 (2.4)	122 (2.5)	305 (3.0)	231 (1.9)	<0.001
**Chronic lung disease**	816 (3.0)	309 (6.2)	365 (3.6)	142 (1.2)	<0.001
**Cancer**	1749 (6.5)	263 (5.3)	766 (7.6)	720 (6.0)	<0.001

Characteristics were measured at the first eligible non-frail interval start. Overall p-values compare the three smoking groups and were calculated using one-way analysis of variance for continuous variables and Pearson chi-squared tests for categorical variables. BMI: body mass index. Cox: Cox proportional hazards. HRS: Health and Retirement Study.

Among 1985 participants who quit smoking during follow-up and had cessation-interval weight-change information, 859 (43.3%) had no weight gain or weight loss, 691 (34.8%) gained 0.1–5.0 kg, 290 (14.6%) gained 5.1–10.0 kg, and 145 (7.3%) gained >10.0 kg. At the first non-smoking wave, mean body weight was 94.5 kg in the >10.0 kg group versus 77.2 kg in the 0.1–5.0 kg group, and obesity prevalence was 64.8% versus 20.8%. Follow-up quitter characteristics and trajectories are shown in Supplementary file eTable 3 and eFigures 2 and 3. Repeated-interval weight-change results are shown in Supplementary file eTable 4.

### Smoking cessation, cessation-interval weight change, and incident frailty

The main Cox results are shown in Table 2. During follow-up, 10641 incident frailty cases occurred over 250439.9 person-years in the overall smoking-status analysis. In the fully adjusted model, past smokers had a lower risk of incident frailty than current smokers (HR=0.82; 95% CI: 0.77–0.87; p<0.001), as did never smokers (HR=0.65; 95% CI: 0.61–0.69; p<0.001). When past smokers were separated by cessation timing, quitters before baseline had lower risk than current smokers (HR=0.79; 95% CI: 0.75–0.84; p<0.001), whereas follow-up quitters overall did not (HR=0.98; 95% CI: 0.90–1.07; p=0.684). Associations differed after follow-up quitters were classified by cessation-interval weight change.

**Table 2:** Associations of time-varying smoking cessation status and weight change across the observed cessation interval with incident frailty, Health and Retirement Study cohort, USA, 1998–2020 (N=27020; 10641 events)

*Smoking status*	*Cases/person-years*	*Model 1* *HR (95% CI); p*	*Model 2* *HR (95% CI); p*	*Model 3* *HR (95% CI); p*
**Panel A. Overall smoking status**				
Current smokers (ref.)	1745/33746.9	1	1	1
Past smokers	4601/102544.7	0.68 (0.64–0.72); <0.001	0.80 (0.76–0.85); <0.001	0.82 (0.77–0.87); <0.001
Never smokers	4295/114148.3	0.57 (0.54–0.60); <0.001	0.65 (0.61–0.69); <0.001	0.65 (0.61–0.69); <0.001
**Panel B. Timing of smoking cessation**				
Current smokers (ref.)	1745/33746.9	1	1	1
Quitters before baseline	3926/89520.0	0.65 (0.61–0.69); <0.001	0.77 (0.73–0.82); <0.001	0.79 (0.75–0.84); <0.001
Follow-up quitters	675/13024.7	0.91 (0.83–1.00); 0.049	0.98 (0.90–1.07); 0.682	0.98 (0.90–1.07); 0.684
Never smokers	4295/114148.3	0.57 (0.53–0.60); <0.001	0.65 (0.61–0.69); <0.001	0.65 (0.61–0.69); <0.001
**Panel C. Follow-up smoking cessation with cessation-interval weight change**				
Current smokers (ref.)	1745/33746.9	1	1	1
Quitters before baseline	3926/89520.0	0.66 (0.63–0.70); <0.001	0.81 (0.76–0.85); <0.001	0.83 (0.78–0.88); <0.001
Follow-up quitters (≤0 kg)	266/5001.9	0.89 (0.79–1.01); 0.078	0.96 (0.84–1.08); 0.478	0.97 (0.86–1.10); 0.646
Follow-up quitter (0.1–5.0 kg)	215/4567.1	0.77 (0.67–0.89); <0.001	0.86 (0.74–0.99); 0.031	0.85 (0.74–0.98); 0.029
Follow-up quitters (5.1–10.0 kg)	92/1956.2	0.91 (0.74–1.12); 0.350	0.98 (0.79–1.20); 0.826	0.97 (0.79–1.19); 0.766
Follow-up quitters (>10.0 kg)	65/833.2	1.60 (1.25–2.04); <0.001	1.65 (1.29–2.10); <0.001	1.59 (1.25–2.03); <0.001
Never smokers	4295/114148.3	0.57 (0.54–0.60); <0.001	0.66 (0.63–0.70); <0.001	0.66 (0.62–0.70); <0.001
**Panel D. Cessation-interval weight change among follow-up quitters**				
Follow-up quitters (0.1–5.0 kg) (ref.)	215/4567.1	1	1	1
Follow-up quitters (≤0 kg)	266/5001.9	1.11 (0.92–1.34); 0.275	1.10 (0.91–1.33); 0.338	1.10 (0.91–1.33); 0.325
Follow-up quitters (5.1–10.0 kg)	92/1956.2	1.16 (0.90–1.50); 0.244	1.16 (0.90–1.50); 0.252	1.15 (0.89–1.49); 0.289
Follow-up quitters (>10.0 kg)	65/833.2	2.05 (1.53–2.74); <0.001	1.99 (1.48–2.68); <0.001	1.94 (1.44–2.61); <0.001

HR: hazard ratio. P-values from time-varying Cox proportional hazards models. Cases/person-years were calculated within the corresponding risk set for each exposure definition. Model 1 was stratified by 5-year age group and study wave. Model 2 further adjusted for sex, race/ethnicity, education level, marital status, and household income. Model 3 further adjusted for alcohol intake and physical activity. Current smokers were the reference in Panels A–C. Panel D was restricted to follow-up-quitter person-time and used 0.1–5.0 kg as the reference. CI: confidence interval. HRS: Health and Retirement Study.

**Figure 1:**
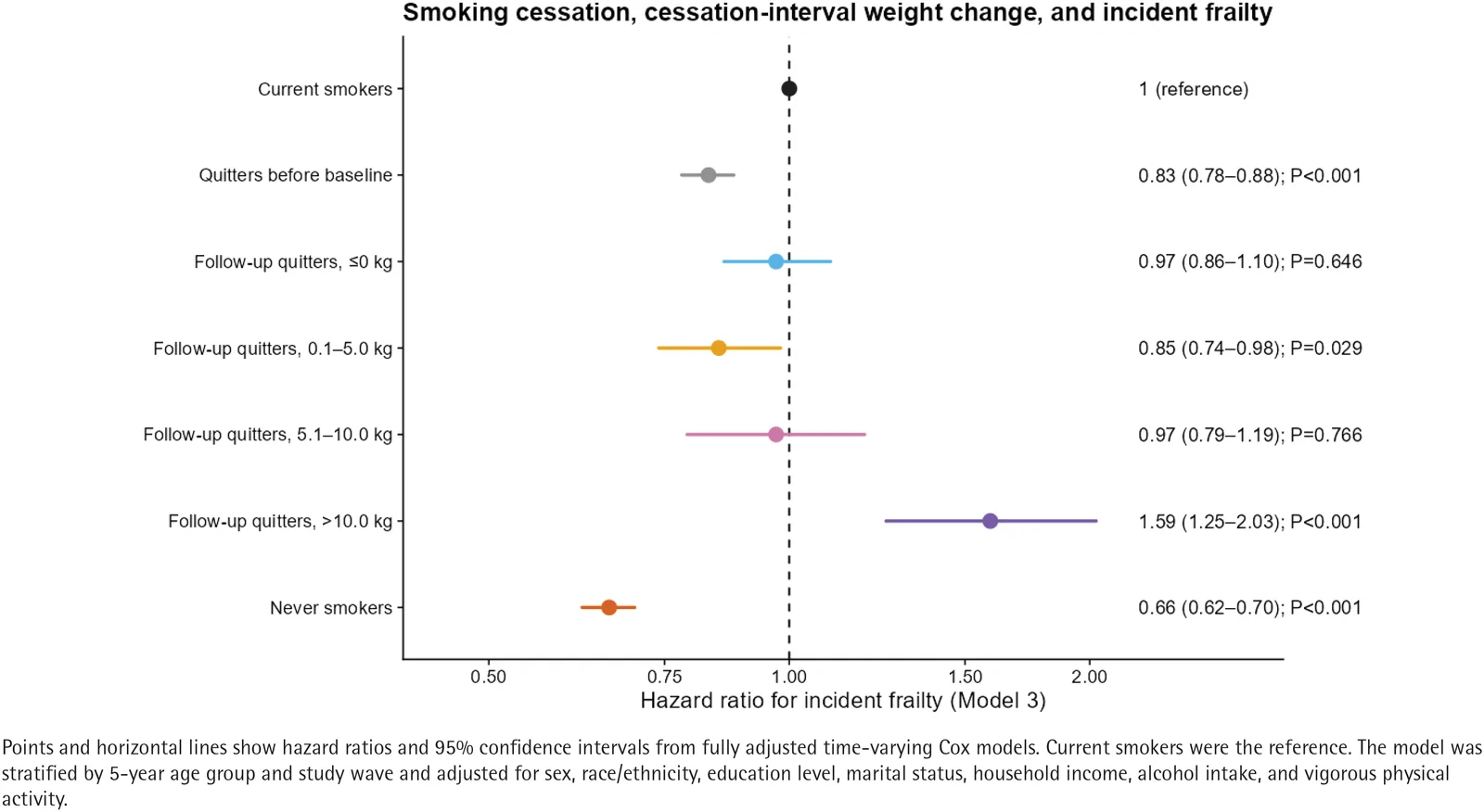
Fully adjusted associations of smoking cessation status and cessation-interval weight change with incident frailty, Health and Retirement Study cohort, USA, 1998–2020 (N=27020; 10641 events)

Compared with current smokers, follow-up quitters who gained 0.1–5.0 kg had lower risk (HR=0.85; 95% CI: 0.74–0.98; p=0.029). Associations were not clear for ≤0 kg (HR=0.97; 95% CI: 0.86–1.10; p=0.646) or 5.1–10.0 kg gain (HR=0.97; 95% CI: 0.79–1.19; p=0.766). Follow-up quitters with >10.0 kg weight gain had a higher risk of incident frailty than current smokers (HR=1.59; 95% CI: 1.25–2.03; p<0.001). Fully adjusted estimates for the primary exposure definition are displayed in Figure 1. In analyses restricted to follow-up quitters, the 0.1–5.0 kg weight-gain group was used as the reference. Compared with this group, quitters with >10.0 kg weight gain had a higher risk of incident frailty (HR=1.94; 95% CI: 1.44–2.61; p<0.001), whereas the ≤0 kg and 5.1–10.0 kg groups did not differ clearly from the reference group.

**Figure 2:**
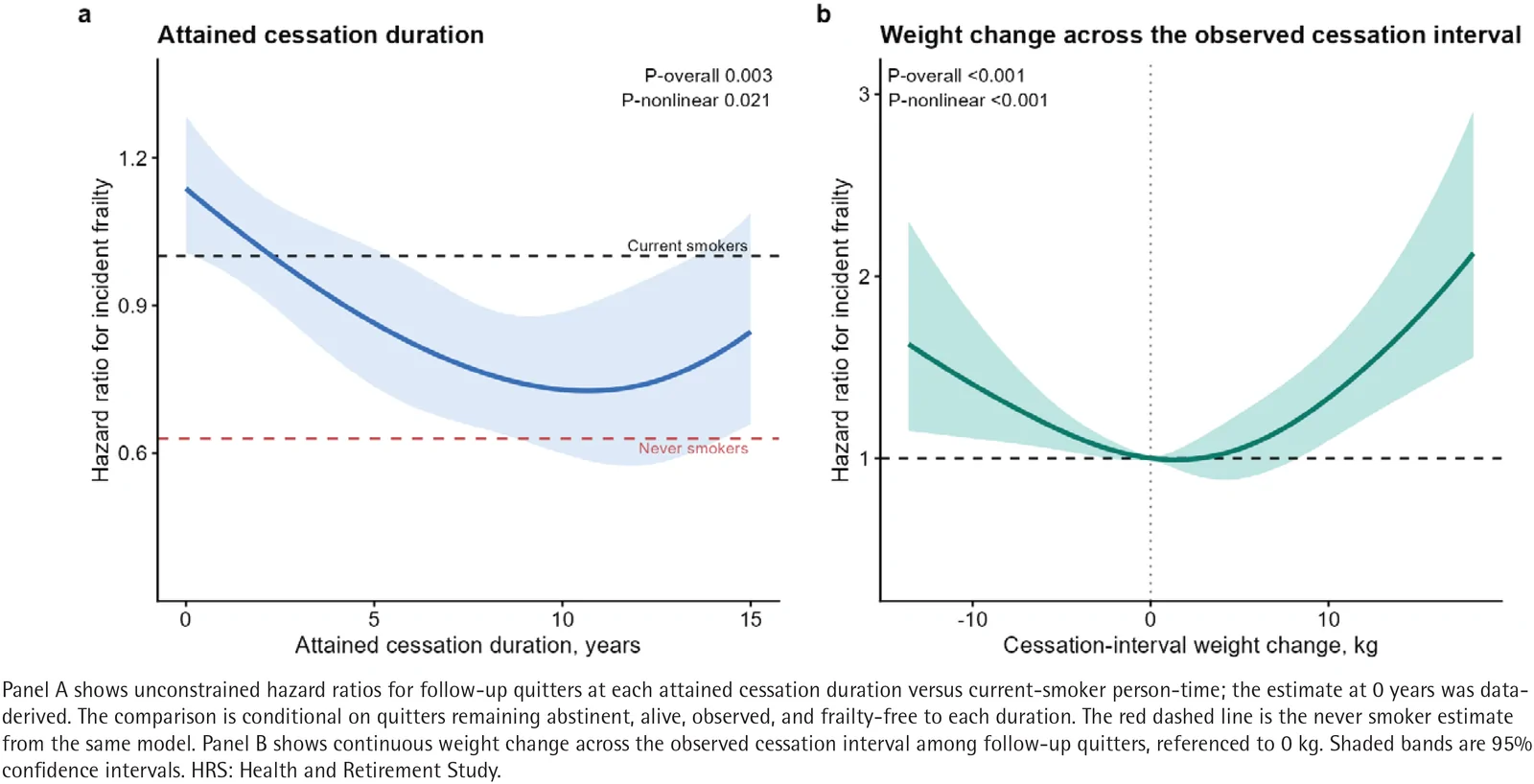
Restricted cubic-spline associations of attained cessation duration and cessation-interval weight change with incident frailty, Health and Retirement Study cohort, USA, 1998–2020 (Panel A: N=27020; 10641 events; Panel B: N=1985 follow-up quitters)

In the duration spline, the HR at 0 years was 1.14 (95% CI: 1.01–1.28), decreasing to 0.79 (95% CI: 0.67–0.93) at 7 years and 0.73 (95% CI: 0.60–0.89) at 10 years (overall p=0.003; nonlinear p=0.021). Cessation-interval weight change showed a nonlinear association with frailty (overall and nonlinear p<0.001). On the gain side, excess risk became more apparent around 9–10 kg; at 10 kg, the HR was 1.34 (95% CI 1.10–1.62) versus 0 kg (Figure 2). The 9–10 kg location should not be interpreted as a biological threshold.

Among eligible analytic follow-up-quitter person-intervals, standardized 2-year risks were 9.5% for 0.1–5.0 kg gain and 15.9% for >10.0 kg gain, a difference of 6.5 percentage points (95% CI: 2.4–10.6), or 65 additional events per 1000 two-year intervals (Supplementary file eTable 7).

### Smoking cessation and annual frailty index change

Frailty-index progression results are shown in Table 3 and Supplementary file eFigure 4. Compared with current smokers, quitters before baseline had slower annual accumulation (β= -0.322 percentage points/year, 95% CI: -0.386 – -0.257; p<0.001). Never smokers also had slower annual frailty index accumulation (β= -0.420; 95% CI: -0.483 – -0.357; p<0.001). Among follow-up quitters, those who gained 0.1–5.0 kg or 5.1–10.0 kg had slower annual frailty index accumulation compared with current smokers. The corresponding β coefficients were -0.220 (95% CI: -0.375 – -0.065; p=0.005) and -0.297 (95% CI: -0.525 – -0.070; p=0.010), respectively. The estimate for the ≤0 kg group was in the same direction, but the 95% CI included the null (β= -0.118; 95% CI: -0.266–0.031; p=0.121). For follow-up quitters with >10.0 kg weight gain, the estimate was close to null and imprecise (β=0.039; 95% CI: -0.302–0.381; p=0.821).

**Table 3:** Associations of time-varying smoking cessation status and weight change across the observed cessation interval with annual frailty-index change, Health and Retirement Study cohort, USA, 1998–2020 (N=27020 overall; N=1985 follow-up quitters)

*Smoking cessation/weight-change group*	*Participants*	*Intervals*	*Model 3* *β (95% CI); p*	*Model 3 + interval-start FI* *β (95% CI); p*
**Panel A. Primary comparison with current smokers**				
Current smokers (ref.)	5463	17074	0	
Quitters before baseline	9721	44958	-0.322 (-0.386 – -0.257); <0.001	
Follow-up quitters (≤0 kg)	926	2491	-0.118 (-0.266–0.031); 0.121	
Follow-up quitters (0.1–5.0 kg)	748	2260	-0.220 (-0.375 – -0.065); 0.005	
Follow-up quitters (5.1–10.0 kg)	317	970	-0.297 (-0.525 – -0.070); 0.010	
Follow-up quitters (>10.0 kg)	154	417	0.039 (-0.302–0.381); 0.821	
Never smokers	12027	57530	-0.420 (-0.483 – -0.357); <0.001	
**Panel B. Direct comparison among follow-up quitters**				
Follow-up quitters (0.1–5.0 kg) (ref.)	748	2260	0	0
Follow-up quitters (≤0 kg)	926	2491	0.125 (-0.080–0.330); 0.231	0.112 (-0.091–0.315); 0.281
Follow-up quitters (5.1–10.0 kg)	317	970	-0.071 (-0.341–0.199); 0.606	-0.066 (-0.334–0.202); 0.632
Follow-up quitters (>10.0 kg)	154	417	0.284 (-0.092–0.660); 0.139	0.415 (0.040–0.790); 0.030

The β coefficients are differences in annual frailty-index change, expressed as percentage points per year. Panel A compares exposure groups with current smokers using Model 3. Panel B directly compares weight-change groups among follow-up quitters using 0.1–5.0 kg gain as the reference, first with Model 3 covariates and then with additional adjustment for the frailty index (FI) measured at interval start. All models adjusted for 5-year age group, study wave, sex, race/ethnicity, education level, marital status, household income, alcohol intake, and vigorous physical activity, with a participant random intercept. Intervals are consecutive interview-to-interview person-intervals. CI: confidence interval. HRS: Health and Retirement Study. Group-specific participant counts are not mutually exclusive because some participants contributed more than one observed cessation episode and weight-change category.

In the direct follow-up-quitter comparison, >10.0 kg versus 0.1–5.0 kg gain corresponded to β=0.284 percentage points/year (95% CI: -0.092–0.660; p=0.139) with Model 3 covariates and β=0.415 (95% CI: 0.040–0.790; p=0.030) after additional adjustment for interval-start frailty index. A sensitivity analysis including participants with baseline frailty is shown in Supplementary file eTable 8.

### Subgroup and sensitivity analyses

Associations varied by age and sex (interaction p=0.010 and p=0.013), but not clearly by BMI or vigorous physical activity (Supplementary file eTable 5). The higher frailty risk associated with >10.0 kg gain was generally observed across subgroups, although estimates were less precise in smaller strata. The sensitivity analyses generally supported the robustness of the main findings (Supplementary file eTable 6), particularly the lower risk among quitters before baseline and never smokers and the higher risk among follow-up quitters with >10.0 kg weight gain. The association for the 0.1–5.0 kg weight-gain group was directionally similar but attenuated in some sensitivity analyses. In the pre-transition BMI/FI complete-case sample, the >10.0 kg versus 0.1–5.0 kg, HR was 1.81 (95% CI: 1.30–2.52) with Model 3 and 1.55 (95% CI: 1.11–2.17) after additional adjustment for pre-transition BMI and frailty index. In the smoking-duration complete-case sample, the corresponding HRs were 2.45 (95% CI: 1.51–3.97) with Model 3 and 2.43 (95% CI: 1.50–3.93) after additional adjustment for pre-cessation smoking duration (Supplementary file eTable 9).

## DISCUSSION

In this longitudinal analysis of middle-aged and older adults, longer attained cessation duration was associated with lower incident-frailty risk. Weight change across the observed cessation interval further distinguished risk among follow-up quitters. Modest gain was not associated with excess frailty risk, whereas substantial gain identified a subgroup with higher relative and absolute risk.

Previous longitudinal studies have established smoking as a risk factor for frailty. A systematic review and cohort analyses linked current smoking with worsening frailty, incident frailty, or greater deficit accumulation^8-11^. In the English Longitudinal Study of Ageing, longer cessation duration was associated with more favorable frailty trajectories, although a gap remained even among participants who had quit at least 41 years earlier^12^. Our attained-duration analysis complements that work by characterizing risk across observed cessation duration. However, estimates at each duration compare current smoker person-time with quitters who remained abstinent, alive, observed, and frailty-free to that duration. These duration-specific estimates are conditional on continued abstinence, survival, observation, and remaining frailty-free, and should not be interpreted as within-person recovery trajectories.

Evidence from cardiometabolic, mortality, and dementia outcomes suggests that weight gain after cessation does not have a uniform health meaning. In the Framingham Offspring Study, weight gain after quitting did not offset the cardiovascular benefit of cessation^23^. Large US cohorts showed a short-term increase in diabetes risk with greater weight gain but persistent cardiovascular and mortality benefits^24^. A recent HRS-based dementia study reported that dementia risk approached the never smoker level after approximately 5–7 years and that benefits were most apparent without substantial weight gain^31^. Our study extends this framework to frailty, a multidomain ageing outcome that reflects cumulative deficits rather than a single disease endpoint.

The continuous curve clarifies the categorical weight-change findings. It showed no abrupt change at 10 kg; instead, risk rose progressively on the gain side and became more apparent around 9–10 kg. Within eligible analytic follow-up-quitter intervals, the standardized 2-year risk difference for >10.0 kg versus 0.1–5.0 kg gain was 6.5 percentage points. Substantial gain is therefore a pragmatic marker for closer follow-up rather than a biological threshold. These absolute-risk estimates pertain to eligible analytic follow-up-quitter intervals and may not generalize to all US quitters. In targeted complete-case analyses, the association was attenuated after adjustment for pre-transition BMI and frailty index, whereas additional adjustment for smoking duration changed it little. Residual confounding nevertheless remains possible.

These findings should not discourage smoking cessation in later life. Rather, they suggest that cessation support for older adults may be strengthened by attention to weight, nutrition, and physical activity. The clinical goal should not be to prevent all weight gain after quitting, because modest gain may be neutral or may accompany improved reserve in some older adults^32^. A more relevant target may be the prevention of substantial gain, particularly when accompanied by reduced activity, cardiometabolic deterioration, or early mobility decline.

### Strengths and limitations

This study has several strengths, including a large longitudinal US cohort, repeated assessments of smoking status, body weight and frailty, and complementary analyses of incident frailty and annual frailty index change.

Several limitations should be noted. First, because this was an observational study, residual confounding and reverse causation cannot be excluded. Although pre-transition frailty index was considered in sensitivity analyses, long-term frailty trajectories and health changes within the cessation interval could not be fully characterized. Second, the exposure captured weight change across an approximately 2-year observed transition, not change exclusively after an exact quit date. The carried-forward category represented the observed cessation episode rather than updated weight status, and later weight trajectories were therefore not captured; intervals after relapse were reclassified as current-smoking person-time. Third, self-reported smoking and weight may introduce information bias and misclassification, and intermittent smoking may have been missed. Fourth, valid consecutive frailty assessments were required; excluded participants were older, frailer, poorer, less active, and more disease-burdened (Supplementary file eTable 1). Fine–Gray models accounted for death as a competing event but did not address nonresponse, loss to follow-up, or analytic-sample selection; observation-probability weighting was not used. Accordingly, absolute-risk estimates pertain to eligible analytic follow-up-quitter intervals and may not generalize to all US quitters. Fifth, some frailty-index deficits may lie on pathways linking smoking and weight change; estimates reflect associations with overall frailty burden rather than single disease-specific endpoint.

## CONCLUSIONS

Longer cessation duration was associated with lower frailty risk. Weight change across the observed cessation interval was nonlinearly associated with frailty, with excess risk becoming more apparent around gains of 9–10 kg without defining a biological threshold. Smoking cessation remains the priority, while substantial gain may warrant continued weight, metabolic, and functional assessment.

## Supplementary Material



## Data Availability

The data used in this study were obtained from the US Health and Retirement Study (HRS). HRS data are publicly available to registered users through the HRS official website: https://hrs.isr.umich.edu/
