# Supplementary material for: Smoking cessation, weight change, and incidence and progression of frailty: A longitudinal analysis of the US Health and Retirement Study
Source: Tob Induc Dis. 2026 Sep 17;24:10.18332/tid/231379. doi: 10.18332/tid/231379 (PMC13584919; doi:10.18332/tid/231379)
Supplement: Supplementary file 1 [file TID-24-167-s1.pdf]

Tobacco Induced Diseases

Supplementary file

© 2026 Liang Y. et al.

DOI:
10.18332/tid/231379

The content has been provided by the author(s) and has not been reviewed, verified, or endorsed by European Publishing. It may not have undergone peer review. The views, opinions, and recommendations expressed are solely those of the author(s) and do not necessarily reflect the position of European Publishing. European Publishing accepts no responsibility or liability for any consequences arising from the use of, or reliance on, this content.

Supplementary Materials

Contents

Supplementary Methods

eMethod 1. Study population and person-period dataset construction

eMethod 2. Frailty index construction

eMethod 3. Definition of smoking cessation and weight change across the observed cessation interval

Supplementary Table S1. Detailed description of the 32-item frailty index used in the Health and Retirement Study cohort, USA, 1998–2020

Supplementary Tables

eTable 1. Characteristics of participants included versus excluded from the incident-frailty analytic sample, Health and Retirement Study cohort, USA, 1998–2020 (N=38403)

eTable 2. Missing or missing/unknown rates of main-analysis variables across eligible person-intervals, Health and Retirement Study cohort, USA, 1998–2020 (125700 person-intervals contributed by N=27020 participants)

eTable 3. Characteristics of follow-up quitters by weight change across the observed cessation interval, Health and Retirement Study cohort, USA, 1998–2020 (N=1985)

eTable 4. Association between time-updated smoking transition status and 2-year body-weight change, Health and Retirement Study cohort, USA, 1998–2020 (105296 person-intervals)

eTable 5. Subgroup analyses of smoking cessation status and cessation-interval weight change with incident frailty, Health and Retirement Study cohort, USA, 1998–2020 (N=27020)

eTable 6. Sensitivity analyses for incident frailty, Health and Retirement Study cohort, USA, 1998–2020 (primary analytic sample N=27020)

eTable 7. Standardized 2-year absolute risk of incident frailty by cessation-interval weight change among eligible analytic follow-up-quitter intervals, Health and Retirement Study cohort, USA, 1998–2020 (N=1985; 6138 intervals; 638 events)

eTable 8. Sensitivity analysis of annual frailty-index change including participants with baseline frailty, Health and Retirement Study cohort, USA, 1998–2020 (group-specific participant and interval counts shown below)

eTable 9. Ssensitivity analyses for incident frailty among follow-up quitters, Health and Retirement Study cohort, USA, 1998–2020 (panel-specific complete-case sample sizes shown below)

Supplementary Methods

eMethod 1. Study population and person-period dataset construction

We used biennial data from the Health and Retirement Study (HRS) from 1998 to 2020. HRS is a nationally representative longitudinal study of U.S. adults older than 50 years, with core interviews conducted every two years. The 1998 wave was selected as the analytic baseline because the original HRS and AHEAD cohorts had been merged by this wave, and the Children of the Depression and War Babies cohorts were added to improve coverage of U.S. adults aged 50 years and older. Subsequent refreshment cohorts entered the study in later waves, including the Early Baby Boomers in 2004, Mid Baby Boomers in 2010, and Late Baby Boomers in 2016. Participants were eligible for the primary incident frailty analysis if a valid consecutive-wave frailty follow-up interval could be constructed, they were non-frail at the interval-start frailty assessment, and smoking status could be classified. Missing categorical covariates were retained as Missing/Unknown categories in the primary analyses rather than used as exclusion criteria.

eMethod 2. Frailty index construction

Frailty was measured using a 32-item deficit-accumulation frailty index. The index included chronic diseases, sensory impairment, self-rated health, activities of daily living, instrumental activities of daily living, mobility and physical function limitations, depressive symptoms, memory, and orientation. Smoking status, body weight, BMI, obesity, and weight loss were not included as frailty index items.

At each wave, the frailty index was calculated as the sum of observed deficit scores divided by the number of nonmissing deficits. A valid frailty index required at least 26 of 32 items to be observed. Continuous or ordinal cognitive components were scaled to the 0–1 deficit range, and binary health or limitation items were coded as 0 for no deficit and 1 for deficit.

For the incident frailty analysis, participants were required to be non-frail at interval start. Incident frailty was defined as frailty index <0.25 at the interval-start wave and frailty index ≥ 0.25 at the next wave. An alternative frailty threshold of frailty index ≥ 0.20 was used in sensitivity analysis.

eMethod 3. Definition of smoking cessation and weight change across the observed cessation interval

At each wave, participants were classified as current smokers, past smokers, or never smokers using current smoking and ever-smoking information. Current smokers were participants reporting current smoking. Past smokers were participants who did not currently smoke but reported ever smoking. Never smokers were participants who did not currently smoke and did not report a history of smoking.

Quitters before baseline were participants whose smoking status at risk-set entry indicated past smoking before the start of observed person-time, as well as past-smoker person-time not observed as a current-to-noncurrent transition during follow-up. Follow-up quitters were participants who transitioned from current smoking at the previous wave to non-current smoking at the current wave.

Weight change across the observed cessation interval was calculated as body weight at the first wave reporting non-smoking minus body weight at the preceding wave. Because the exact quit date was unavailable, this approximately 2-year interval may include weight change before and after cessation. Categories were ≤ 0 kg, 0.1–5.0 kg, 5.1–10.0 kg, and >10.0 kg. The category marked the observed cessation episode and was carried forward during continued non-smoking intervals within that episode; later weight measurements did not reclassify the episode. Intervals after relapse were reclassified as current-smoking person-time.

Supplementary Table S1. Detailed description of the 32-item frailty index used in the Health and Retirement Study cohort, USA, 1998–2020

The table lists the 32 deficits included in the frailty index. Smoking status, body weight, body mass index, obesity, and weight loss were not included.

Domains	Description of the items	Cut-off value
**Medical conditions and self-rated health**	Self-reported physician-diagnosed hypertension	Medical conditions: yes = 1, no = 0; eyesight, hearing, and general health: poor or fair = 1, excellent, very good, or good = 0.
	Self-reported physician-diagnosed diabetes	
	Self-reported physician-diagnosed cancer	
	Self-reported physician-diagnosed chronic lung disease	
	Self-reported physician-diagnosed arthritis	
	Self-reported physician-diagnosed heart disease	
	Self-reported physician-diagnosed stroke	
	Self-reported physician-diagnosed psychiatric problems	
	Self-reported physician-diagnosed memory-related disease	
	Self-reported distant eyesight	

Domains	Description of the items	Cut-off value
	Self-reported near eyesight	
	Self-reported hearing	
	Self-reported general health status	
**ADL/IADL disabilities**	Difficulty with dressing	Yes = 1, no = 0.
	Difficulty with bathing or showering	
	Difficulty with eating	
	Difficulty with getting in or out of bed	
	Difficulty with using the toilet	
	Difficulty with managing money	
	Difficulty with taking medications	
	Difficulty with shopping for groceries	
	Difficulty with preparing meals	
**Functional abilities**	Difficulty with walking one block	Yes = 1, no = 0.
	Difficulty with getting up from a chair after sitting for long periods	
	Difficulty with climbing several flights of stairs	
	Difficulty with lifting or carrying weights	
	Difficulty with picking up a dime from the table	
	Difficulty with stooping, kneeling, or crouching	
	Difficulty with reaching or extending arms	
**Depressive symptoms and cognition**	CES-D depressive symptom score	CES-D score ≥4 = 1, <4 = 0; memory and orientation scores were scaled to the 0–1 deficit range.
	Word recall memory score	
	Orientation to month, day, year, and day of week	

Notes: FI, frailty index; HRS, Health and Retirement Study; ADL, activities of daily living; IADL, instrumental activities of daily living; CES-D, Center for Epidemiologic Studies Depression Scale. Heart disease included angina, coronary heart disease, congestive heart failure, myocardial infarction, or other heart problems. Memory-related disease included memory disease, Alzheimer disease, dementia, or other serious memory impairment. A valid FI required at least 26 of 32 items.

Supplementary Tables

eTable 1. Characteristics of participants included versus excluded from the incident-frailty analytic sample, Health and Retirement Study cohort, USA, 1998–2020 (N=38403)

Characteristics	Included (N = 27020)	Excluded (N = 11383)	Standardized mean difference
**Age, years, mean (SD)**	59.6 (10.1)	64.1 (12.8)	0.390
**Sex, n (%)**			
Male	12115 (44.8)	4585 (40.3)	0.092
Female	14905 (55.2)	6798 (59.7)	0.092
**Race/ethnicity, n (%)**			
White	18130 (67.1)	6509 (57.2)	0.205
Black	4554 (16.9)	2696 (23.7)	0.171
Other	4318 (16.0)	2167 (19.1)	0.081
**Education, n (%)**			
Less than high school	4902 (18.1)	4141 (36.4)	0.419
GED	1225 (4.5)	697 (6.1)	0.071
High school graduate	7769 (28.8)	3026 (26.6)	0.048
Some college	6671 (24.7)	2265 (19.9)	0.115
College and above	6450 (23.9)	1242 (10.9)	0.347
**Marital status, n (%)**			
Married/partnered	19947 (73.9)	6677 (58.8)	0.324
Not married/partnered	7053 (26.1)	4684 (41.2)	0.324
**Household income, n (%)**			
<$20000	5703 (21.1)	5341 (46.9)	0.566
$20000–<40000	6261 (23.2)	2714 (23.8)	0.016
$40000–<80000	7451 (27.6)	1951 (17.1)	0.252
≥$80000	7605 (28.1)	1377 (12.1)	0.409
**Body weight, kg, mean (SD)**	79.4 (17.9)	80.6 (21.4)	0.061
**BMI category, kg/m², n (%)**			
<25.0	8670 (32.7)	3561 (32.1)	0.014
25.0–29.9	10617 (40.0)	3606 (32.5)	0.158
≥30.0	7225 (27.3)	3939 (35.5)	0.178
**Frailty index, mean (SD)**	0.1 (0.1)	0.3 (0.2)	1.740
**Valid frailty index items, mean (SD)**	31.5 (0.9)	31.0 (2.0)	0.322
**Vigorous physical activity, n (%)**			
<3 times/week	15314 (56.7)	9076 (79.8)	0.513
≥3 times/week	11681 (43.3)	2291 (20.2)	0.513

Characteristics	Included (N = 27020)	Excluded (N = 11383)	Standardized mean difference
**Alcohol intake, n (%)**			
Non-drinker	16122 (59.9)	8540 (75.4)	0.337
≤1 drink/day	7537 (28.0)	1878 (16.6)	0.277
1–2 drinks/day	2009 (7.5)	511 (4.5)	0.124
>2 drinks/day	1258 (4.7)	392 (3.5)	0.061
**Diabetes, n (%)**	2715 (10.0)	2979 (26.2)	0.428
**Heart disease, n (%)**	2718 (10.1)	3470 (30.5)	0.525
**Stroke, n (%)**	658 (2.4)	1593 (14.0)	0.431
**Chronic lung disease, n (%)**	816 (3.0)	1642 (14.4)	0.413
**Cancer, n (%)**	1749 (6.5)	1478 (13.0)	0.221

Values are mean (standard deviation) for continuous variables and n (%) for categorical variables. Included participants formed the participant-level sample used in main Table 1. Excluded participants were source participants who did not enter the incident-frailty analytic sample. Percentages were calculated among participants with available data. Standardized mean differences describe group differences. BMI, body mass index; GED, General Educational Development; HRS, Health and Retirement Study. SD, standard deviation.

eTable 2. Missing or missing/unknown rates of main-analysis variables across eligible person-intervals, Health and Retirement Study cohort, USA, 1998–2020 (125700 person-intervals contributed by N=27020 participants)

Variable	Missing/Unknown person-intervals, n	Missing/Unknown person-intervals, %
**Demographic and socioeconomic covariates**		
Age group	0	0.0
Study wave	0	0.0
Sex	0	0.0
Race/ethnicity	47	<0.1
Education	20	<0.1
Marital status	82	0.1
Household income category	0	0.0
**Behavioural covariates**		
Alcohol intake	427	0.3
Physical activity	219	0.2
**Outcome variables**		
Incident frailty	0	0.0
Follow-up time	0	0.0

Percentages were calculated across 125700 eligible person-intervals. Values are n (%). Missing categorical covariate values were retained as a Missing/Unknown category in the primary analysis. HRS, Health and Retirement Study.

eTable 3. Characteristics of follow-up quitters by weight change across the observed cessation interval, Health and Retirement Study cohort, USA, 1998–2020 (N=1985)

Characteristics	All follow-up quitters (N = 1985)	Follow-up quitters, ≤0 kg (N = 859)	Follow-up quitters, 0.1–5.0 kg (N = 691)	Follow-up quitters, 5.1–10.0 kg (N = 290)	Follow-up quitters, >10.0 kg (N = 145)
**Age, years, mean (SD)**	62.2 (8.7)	62.5 (8.9)	62.4 (8.6)	61.0 (8.9)	61.2 (7.5)
**Sex, n (%)**					
Male	979 (49.3)	432 (50.3)	329 (47.6)	143 (49.3)	75 (51.7)
Female	1006 (50.7)	427 (49.7)	362 (52.4)	147 (50.7)	70 (48.3)
**Race/ethnicity, n (%)**					
White	1325 (66.8)	511 (59.5)	497 (71.9)	212 (73.1)	105 (72.4)
Black	386 (19.4)	207 (24.1)	104 (15.1)	46 (15.9)	29 (20.0)
Other	274 (13.8)	141 (16.4)	90 (13.0)	32 (11.0)	11 (7.6)
**Education, n (%)**					

Characteristics	All follow-up quitters (N = 1985)	Follow-up quitters, ≤0 kg (N = 859)	Follow-up quitters, 0.1–5.0 kg (N = 691)	Follow-up quitters, 5.1–10.0 kg (N = 290)	Follow-up quitters, >10.0 kg (N = 145)
Less than high school	380 (19.1)	172 (20.0)	126 (18.2)	59 (20.3)	23 (15.9)
GED	103 (5.2)	36 (4.2)	38 (5.5)	18 (6.2)	11 (7.6)
High school graduate	619 (31.2)	266 (31.0)	214 (31.0)	78 (26.9)	61 (42.1)
Some college	566 (28.5)	258 (30.0)	191 (27.6)	80 (27.6)	37 (25.5)
College and above	316 (15.9)	126 (14.7)	122 (17.7)	55 (19.0)	13 (9.0)
**Household income, n (%)**					
<$20000	468 (23.6)	231 (26.9)	149 (21.6)	54 (18.6)	34 (23.4)
$20000–<40000	480 (24.2)	215 (25.0)	164 (23.7)	73 (25.2)	28 (19.3)
$40000–<80000	569 (28.7)	228 (26.5)	207 (30.0)	90 (31.0)	44 (30.3)
≥$80000	468 (23.6)	185 (21.5)	171 (24.7)	73 (25.2)	39 (26.9)
**Body weight, kg, mean (SD)**	78.9 (17.2)	75.4 (16.3)	77.2 (15.6)	85.6 (16.9)	94.5 (17.6)
**BMI category, kg/m², n (%)**					
<25.0	709 (35.7)	399 (46.4)	252 (36.5)	47 (16.2)	11 (7.6)
25.0–29.9	764 (38.5)	306 (35.6)	295 (42.7)	123 (42.4)	40 (27.6)
≥30.0	510 (25.7)	152 (17.7)	144 (20.8)	120 (41.4)	94 (64.8)
**Frailty index, mean (SD)**	0.1 (0.1)	0.1 (0.1)	0.1 (0.1)	0.1 (0.1)	0.1 (0.1)
**Vigorous physical activity, n (%)**					
<3 times/week	1319 (66.4)	560 (65.2)	445 (64.4)	211 (72.8)	103 (71.0)
≥3 times/week	662 (33.4)	296 (34.5)	246 (35.6)	78 (26.9)	42 (29.0)
**Alcohol intake, n (%)**					
Non-drinker	1121 (56.5)	495 (57.6)	388 (56.2)	148 (51.0)	90 (62.1)
≤1 drink/day	574 (28.9)	254 (29.6)	193 (27.9)	98 (33.8)	29 (20.0)
1–2 drinks/day	189 (9.5)	74 (8.6)	68 (9.8)	30 (10.3)	17 (11.7)
>2 drinks/day	95 (4.8)	33 (3.8)	39 (5.6)	14 (4.8)	9 (6.2)
**Diabetes, n (%)**	245 (12.3)	120 (14.0)	83 (12.0)	24 (8.3)	18 (12.4)
**Heart disease, n (%)**	271 (13.7)	117 (13.6)	93 (13.5)	36 (12.4)	25 (17.2)
**Stroke, n (%)**	82 (4.1)	41 (4.8)	31 (4.5)	7 (2.4)	3 (2.1)

Characteristics	All follow-up quitters (N = 1985)	Follow-up quitters, ≤0 kg (N = 859)	Follow-up quitters, 0.1–5.0 kg (N = 691)	Follow-up quitters, 5.1–10.0 kg (N = 290)	Follow-up quitters, >10.0 kg (N = 145)
**Chronic lung disease, n (%)**	201 (10.1)	81 (9.4)	70 (10.1)	29 (10.0)	21 (14.5)
**Cancer, n (%)**	181 (9.1)	86 (10.0)	51 (7.4)	28 (9.7)	16 (11.0)

Values are mean (standard deviation) or n (%). Follow-up quitters were current smokers at the preceding wave and non-current smokers at the current wave. Weight change was body weight at the first wave reporting non-smoking minus body weight at the preceding wave. BMI, body mass index; HRS, Health and Retirement Study. SD, standard deviation.

eTable 4. Association between time-updated smoking transition status and 2-year body-weight change, Health and Retirement Study cohort, USA, 1998–2020 (105296 person-intervals)

Smoking transition status	Participants	Intervals	β (95% CI), kg	P value
Continuing smokers	4007	12183	0 (reference)	—
Follow-up quitters	1966	2245	1.790 (1.553 to 2.027)	<0.001
Quitters before baseline	9674	42425	0.427 (0.317 to 0.537)	<0.001
Never smokers	10777	48443	0.353 (0.243 to 0.462)	<0.001

β coefficients (95% confidence intervals) are adjusted differences in 2-year body-weight change, expressed in kilograms, compared with continuing smokers. The linear mixed-effects model adjusted for 5-year age group, study wave, sex, race/ethnicity, education, marital status, household income, alcohol intake, and vigorous physical activity, with a participant random intercept. CI, confidence interval; HRS, Health and Retirement Study.

eTable 5. Subgroup analyses of smoking cessation status and cessation-interval weight change with incident frailty, Health and Retirement Study cohort, USA, 1998–2020 (N=27020)

Subgroup	Smoking status	Cases/person-years	HR (95% CI)	P value	P for interaction
**Age, years**					0.010
<70	Current smokers	1314/28319.9	1 (reference)	-	
	Quitters before baseline	1547/55964.0	0.78 (0.73–0.85)	<0.001	
	Follow-up quitters, ≤0 kg	120/3240.2	0.97 (0.80–1.17)	0.728	
	Follow-up quitters, 0.1–5.0 kg	107/2905.7	1.01 (0.83–1.23)	0.911	
	Follow-up quitters, 5.1–10.0 kg	44/1382.3	0.90 (0.67–1.22)	0.512	
	Follow-up quitters, >10.0 kg	37/561.0	1.59 (1.14–2.21)	0.006	
	Never smokers	1823/76034.3	0.63 (0.58–0.68)	<0.001	
≥70	Current smokers	431/5427.0	1 (reference)	-	
	Quitters before baseline	2378/33525.5	0.86 (0.78–0.96)	0.007	
	Follow-up quitters, ≤0 kg	146/1761.7	1.12 (0.93–1.36)	0.225	
	Follow-up quitters, 0.1–5.0 kg	108/1661.3	0.88 (0.71–1.09)	0.246	
	Follow-up quitters, 5.1–10.0 kg	48/573.8	1.33 (0.98–1.79)	0.066	
	Follow-up quitters, >10.0 kg	28/272.2	2.01 (1.37–2.96)	<0.001	
	Never smokers	2472/38113.9	0.73 (0.66–0.81)	<0.001	
**Sex**					0.013
Male	Current smokers	791/15743.2	1 (reference)	-	
	Quitters before baseline	2199/48593.2	0.79 (0.73–0.87)	<0.001	
	Follow-up quitters, ≤0 kg	125/2434.9	0.93 (0.77–1.13)	0.482	
	Follow-up quitters, 0.1–5.0 kg	104/2031.1	1.09 (0.88–1.34)	0.437	
	Follow-up quitters, 5.1–10.0 kg	48/1008.2	1.15 (0.86–1.54)	0.355	
	Follow-up quitters, >10.0 kg	33/422.5	1.63 (1.14–2.32)	0.007	

Subgroup	Smoking status	Cases/person-years	HR (95% CI)	P value	P for interaction
	Never smokers	1167/38345.4	0.62 (0.56–0.68)	<0.001	
Female	Current smokers	953/18002.2	1 (reference)	-	
	Quitters before baseline	1726/40896.2	0.78 (0.71–0.84)	<0.001	
	Follow-up quitters, ≤0 kg	141/2567.0	1.04 (0.87–1.25)	0.640	
	Follow-up quitters, 0.1–5.0 kg	111/2536.0	0.76 (0.63–0.93)	0.009	
	Follow-up quitters, 5.1–10.0 kg	44/947.9	0.93 (0.68–1.26)	0.633	
	Follow-up quitters, >10.0 kg	32/410.8	1.81 (1.27–2.59)	0.001	
	Never smokers	3128/75802.8	0.65 (0.61–0.71)	<0.001	
**BMI, kg/m²**					0.647
<25.0	Current smokers	650/14134.2	1 (reference)	-	
	Quitters before baseline	1069/25582.8	0.68 (0.61–0.75)	<0.001	
	Follow-up quitters, ≤0 kg	117/2172.0	1.04 (0.85–1.28)	0.683	
	Follow-up quitters, 0.1–5.0 kg	65/1440.5	0.81 (0.62–1.06)	0.124	
	Follow-up quitters, 5.1–10.0 kg	19/358.2	1.22 (0.77–1.93)	0.401	
	Follow-up quitters, >10.0 kg	6/70.7	2.21 (0.97–5.04)	0.060	
	Never smokers	1375/37256.0	0.57 (0.52–0.64)	<0.001	
≥25.0	Current smokers	1071/19219.7	1 (reference)	-	
	Quitters before baseline	2794/62914.7	0.77 (0.72–0.83)	<0.001	
	Follow-up quitters, ≤0 kg	148/2808.9	0.94 (0.79–1.12)	0.501	
	Follow-up quitters, 0.1–5.0 kg	150/3119.5	0.89 (0.75–1.05)	0.175	
	Follow-up quitters, 5.1–10.0 kg	73/1596.0	0.92 (0.73–1.17)	0.520	
	Follow-up quitters, >10.0 kg	59/762.6	1.50 (1.15–1.96)	0.003	
	Never smokers	2824/75032.5	0.63 (0.59–0.68)	<0.001	

Subgroup	Smoking status	Cases/person-years	HR (95% CI)	P value	P for interaction
**Vigorous physical activity**					0.533
<3 times/week	Current smokers	1358/23294.2	1 (reference)	-	
	Quitters before baseline	2863/52475.7	0.80 (0.75–0.86)	<0.001	
	Follow-up quitters, ≤0 kg	208/3372.0	1.00 (0.86–1.16)	0.964	
	Follow-up quitters, 0.1–5.0 kg	175/3092.2	0.91 (0.77–1.06)	0.228	
	Follow-up quitters, 5.1–10.0 kg	77/1421.3	1.10 (0.88–1.39)	0.398	
	Follow-up quitters, >10.0 kg	47/608.0	1.50 (1.12–2.02)	0.006	
	Never smokers	3273/71178.2	0.66 (0.62–0.71)	<0.001	
≥3 times/week	Current smokers	383/10401.0	1 (reference)	-	
	Quitters before baseline	1047/36868.1	0.79 (0.70–0.90)	<0.001	
	Follow-up quitters, ≤0 kg	58/1611.8	1.04 (0.79–1.38)	0.775	
	Follow-up quitters, 0.1–5.0 kg	40/1474.8	0.86 (0.62–1.20)	0.368	
	Follow-up quitters, 5.1–10.0 kg	15/530.5	0.87 (0.52–1.47)	0.607	
	Follow-up quitters, >10.0 kg	18/225.2	2.42 (1.49–3.91)	<0.001	
	Never smokers	1012/42782.8	0.61 (0.54–0.69)	<0.001	

Values are hazard ratios (95% confidence intervals) from Model 3 Cox models within each subgroup. Current smokers were the reference. Model 3 adjusted for sex, race/ethnicity, education, marital status, household income, alcohol intake, and vigorous physical activity, with stratification by 5-year age group and study wave where appropriate. A subgroup-defining covariate was omitted from that subgroup model. Interaction P values used likelihood-ratio tests. BMI, body mass index; CI, confidence interval; HR, hazard ratio; HRS, Health and Retirement Study.

eTable 6. Sensitivity analyses for incident frailty, Health and Retirement Study cohort, USA, 1998–2020 (primary analytic sample N=27020)

Smoking status	Primary analysis	Alternative FI threshold, FI ≥0.20	Excluding events within first 2 years	Excluding baseline major chronic diseases	Fine-Gray competing risk	MICE sensitivity
Current smokers	1 (reference)	1 (reference)	1 (reference)	1 (reference)	1 (reference)	1 (reference)
Quitters before baseline	0.83 (0.78–0.88)	0.89 (0.84–0.94)	0.80 (0.74–0.85)	0.83 (0.77–0.90)	0.76 (0.72–0.81)	0.83 (0.78–0.87)
Follow-up quitters, ≤0 kg	0.97 (0.86–1.10)	1.03 (0.91–1.16)	1.04 (0.91–1.19)	1.03 (0.86–1.24)	0.96 (0.74–1.26)	0.96 (0.85–1.09)
Follow-up quitters, 0.1–5.0 kg	0.85 (0.74–0.98)	0.96 (0.84–1.09)	0.92 (0.79–1.07)	0.80 (0.64–0.98)	0.80 (0.58–1.12)	0.85 (0.74–0.98)
Follow-up quitters, 5.1–10.0 kg	0.97 (0.79–1.19)	1.08 (0.88–1.32)	1.08 (0.86–1.34)	1.05 (0.77–1.43)	0.60 (0.34–1.06)	0.96 (0.78–1.18)
Follow-up quitters, >10.0 kg	1.59 (1.25–2.03)	1.44 (1.10–1.88)	1.69 (1.30–2.19)	1.49 (1.01–2.20)	1.38 (1.08–2.18)	1.59 (1.24–2.02)
Never smokers	0.66 (0.62–0.70)	0.72 (0.68–0.76)	0.64 (0.60–0.69)	0.64 (0.59–0.70)	0.61 (0.58–0.65)	0.66 (0.62–0.69)

Values are hazard ratios (95% confidence intervals) unless indicated. Cox sensitivity analyses used the Model 3 adjustment set, with stratification by 5-year age group and study wave and adjustment for sex, race/ethnicity, education, marital status, household income, alcohol intake, and vigorous physical activity. The Fine–Gray analysis was a participant-level model using entry exposure and covariate values and adjusted for sex, race/ethnicity, education, marital status, household income, alcohol intake, and vigorous physical activity; it reports subdistribution hazard ratios for incident frailty, with death treated as a competing event. Current smokers were the reference. CI, confidence interval; FI, frailty index; HRS, Health and Retirement Study; MICE, multiple imputation by chained equations.

eTable 7. Standardized 2-year absolute risk of incident frailty by cessation-interval weight change among eligible analytic follow-up-quitter intervals, Health and Retirement Study cohort, USA, 1998–2020 (N=1985; 6138 intervals; 638 events)

Weight change	Cases/ intervals	Standardized 2-year risk, % (95% CI)	Risk difference, percentage points (95% CI)	Excess events per 1000 intervals (95% CI)	Standardized risk ratio (95% CI)	P value
0.1–5.0 kg	215/2260	9.5 (8.3 to 10.7)	Reference	Reference	1.00 (Reference)	Reference
≤0 kg	266/2491	10.4 (9.3 to 11.6)	1.0 (-0.7 to 2.7)	10 (-7 to 27)	1.10 (0.93 to 1.31)	0.253
5.1–10.0 kg	92/970	10.0 (8.0 to 11.9)	0.5 (-1.8 to 2.8)	5 (-18 to 28)	1.05 (0.83 to 1.33)	0.670
>10.0 kg	65/417	15.9 (12.0 to 19.9)	6.5 (2.4 to 10.6)	65 (24 to 106)	1.69 (1.28 to 2.23)	0.002

Values are model-standardized estimates (95% confidence intervals). Risks were standardized only to the covariate distribution of eligible follow-up-quitter person-intervals in the analytic sample. The pooled complementary log-log model used log(interval length/2 years) as an offset and participant-clustered robust standard errors and adjusted for 5-year age group, study wave, sex, race/ethnicity, education, marital status, household income, alcohol intake, and vigorous physical activity. The 0.1–5.0 kg group was the reference. Overall Wald P=0.004. Excess events are per 1000 two-year person-intervals. CI, confidence interval; HRS, Health and Retirement Study.

eTable 8. Sensitivity analysis of annual frailty-index change including participants with baseline frailty, Health and Retirement Study cohort, USA, 1998–2020 (N=34,194)

Smoking status	Participants	Intervals	Difference in annual FI change, β (95% CI), percentage points/year	P value
Current smokers	7484	26604	0 (reference)	—
Quitters before baseline	12237	65259	-0.300 (-0.365 to -0.234)	<0.001
Follow-up quitters, ≤0 kg	1621	4525	-0.159 (-0.299 to -0.020)	0.025
Follow-up quitters, 0.1–5.0 kg	1163	3807	-0.272 (-0.422 to -0.121)	<0.001
Follow-up quitters, 5.1–10.0 kg	541	1786	-0.402 (-0.613 to -0.190)	<0.001
Follow-up quitters, >10.0 kg	307	1025	-0.154 (-0.429 to 0.121)	0.271
Never smokers	14565	80182	-0.353 (-0.418 to -0.288)	<0.001

β coefficients (95% confidence intervals) are differences in annual frailty-index change, expressed as percentage points per year, compared with current smokers. The linear mixed-effects model adjusted for 5-year age group, study wave, sex, race/ethnicity, education, marital status, household income, alcohol intake, and vigorous physical activity, with a participant random intercept. This sensitivity analysis included participants with baseline frailty. CI, confidence interval; FI, frailty index; HRS, Health and Retirement Study.

eTable 9. Sensitivity analyses for incident frailty among follow-up quitters, Health and Retirement Study cohort, USA, 1998–2020 (Panel A: N=1662; Panel B: N=1112)

Model	Weight-change group	Participants	Intervals	Events	HR (95% CI)	P value
**Panel A. Additional adjustment for pre-transition BMI and FI**						
Model 3	0.1–5.0 kg	618	1730	168	1.00 (reference)	—
	≤0 kg	788	2049	224	1.06 (0.86–1.32)	0.576
	5.1–10.0 kg	260	778	77	1.18 (0.89–1.57)	0.260
	>10.0 kg	127	337	54	1.81 (1.30–2.52)	<0.001
Model 3 + pre-transition BMI + FI	0.1–5.0 kg	618	1730	168	1.00 (reference)	—
	≤0 kg	788	2049	224	0.91 (0.73–1.14)	0.414
	5.1–10.0 kg	260	778	77	1.07 (0.80–1.42)	0.657
	>10.0 kg	127	337	54	1.55 (1.11–2.17)	0.010
**Panel B. Additional adjustment for pre-cessation smoking duration**						
Model 3	0.1–5.0 kg	398	1007	82	1.00 (reference)	—
	≤0 kg	531	1261	127	1.17 (0.87–1.57)	0.312
	5.1–10.0 kg	173	481	50	1.46 (1.00–2.15)	0.051
	>10.0 kg	78	166	28	2.45 (1.51–3.97)	<0.001
Model 3 + smoking duration	0.1–5.0 kg	398	1007	82	1.00 (reference)	—
	≤0 kg	531	1261	127	1.15 (0.85–1.55)	0.369
	5.1–10.0 kg	173	481	50	1.47 (1.00–2.16)	0.048
	>10.0 kg	78	166	28	2.43 (1.50–3.93)	<0.001

All comparisons used 0.1–5.0 kg gain as the reference. Within each panel, Model 3 and the additionally adjusted model were estimated in the same complete-case person-intervals. Pre-transition BMI and FI were measured at the wave preceding the observed cessation transition. Pre-cessation smoking duration was calculated as age at that preceding smoking wave minus self-reported age at smoking initiation. Model 3 was stratified by 5-year age group and study wave and adjusted for sex, race/ethnicity, education, marital status, household income, alcohol intake, and vigorous physical activity. Group-specific participant counts are not mutually exclusive because some participants contributed more than one observed cessation episode and weight-change category. BMI, body mass index; CI, confidence interval; FI, frailty index; HR, hazard ratio.

Supplementary Figures

eFigure 1. Study-population inclusion flow chart, Health and Retirement Study cohort, USA, 1998–2020 (source population N=38403; final analytic sample N=27020)

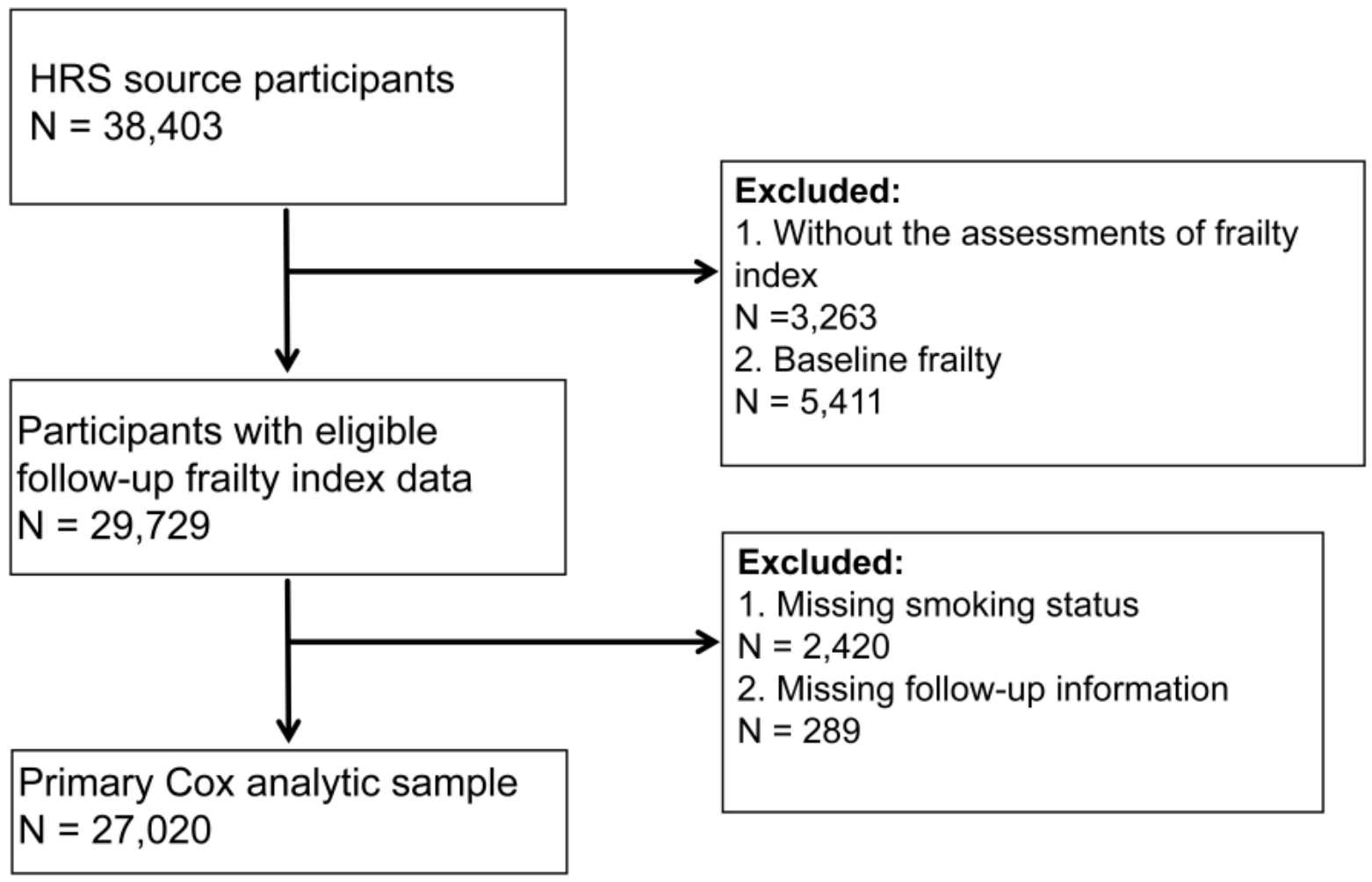

HRS, Health and Retirement Study.

eFigure 2. Mean body weight and 2-year weight change around the observed cessation transition by cessation-interval weight-change group, Health and Retirement Study cohort, USA, 1998–2020 (N=1985 follow-up quitters)

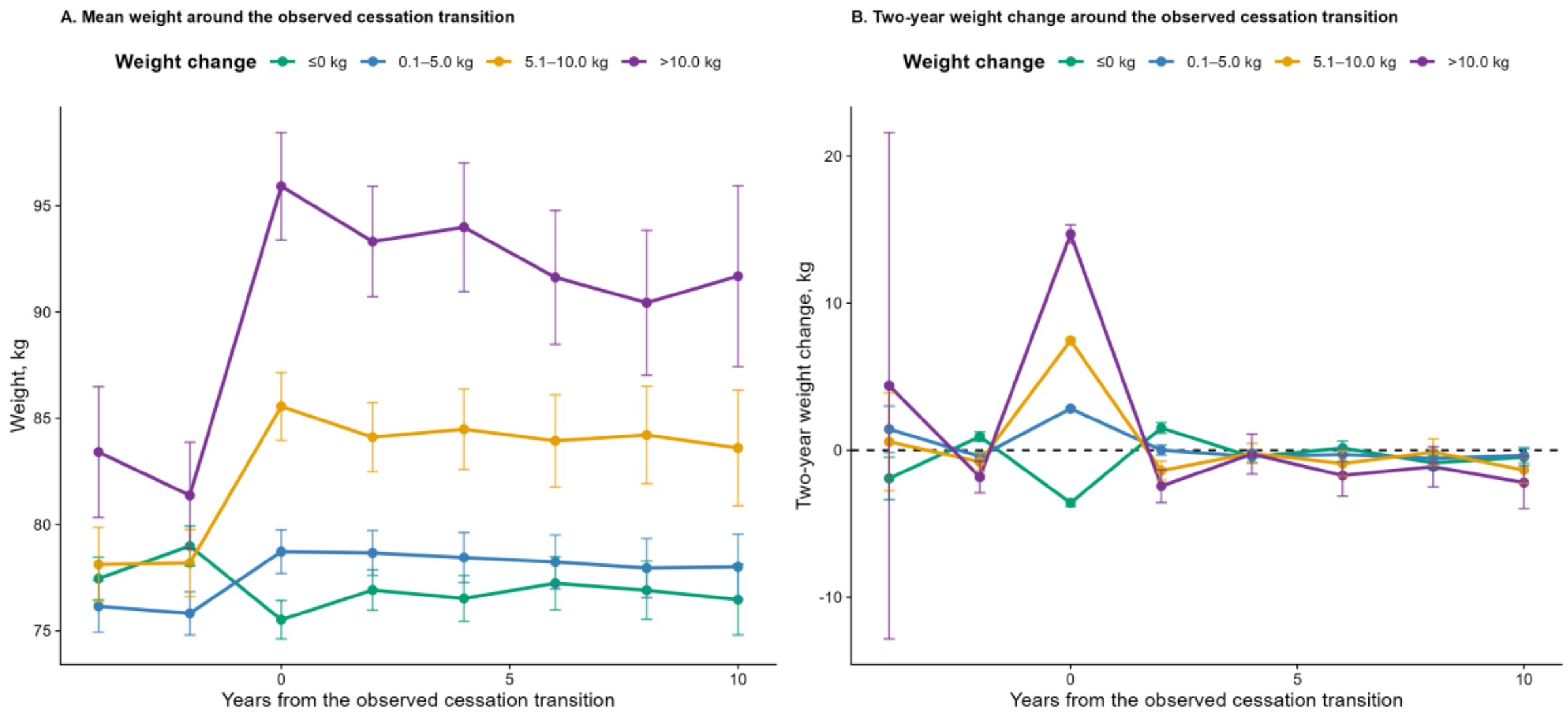

Points show means; thin vertical lines show 95% confidence intervals for the mean. The observed transition is the first wave reporting non-smoking after a current-smoking wave; exact quit dates were unavailable. HRS, Health and Retirement Study.

eFigure 3. Mean body-weight trajectories by smoking pattern, Health and Retirement Study cohort, USA, 1998–2020 (N=17830 participants)

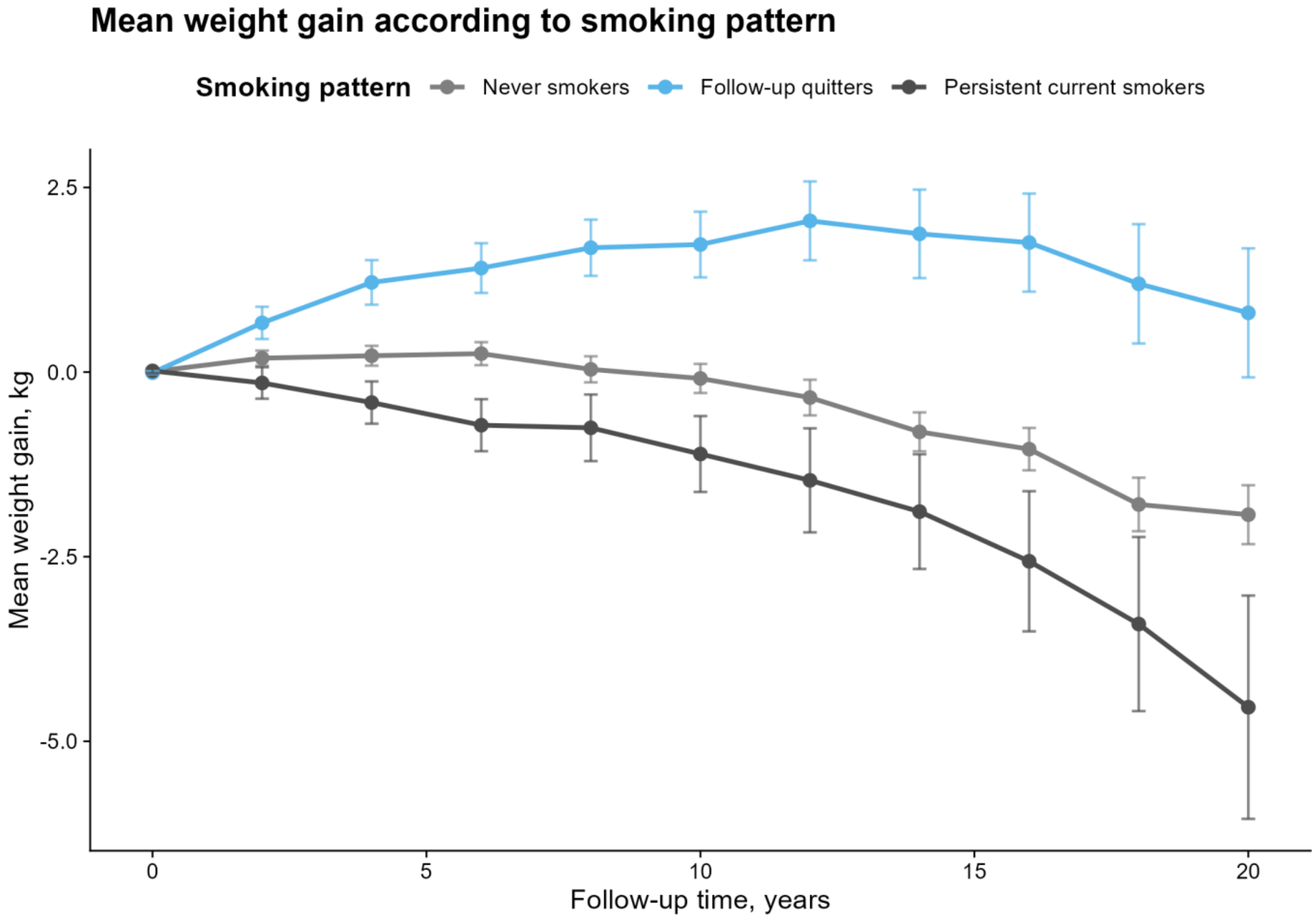

Points show mean weight change from analytic entry; thin vertical lines show 95% confidence intervals for the mean. HRS, Health and Retirement Study.

eFigure 4. Associations of smoking cessation status and cessation-interval weight change with annual frailty-index change, Health and Retirement Study cohort, USA, 1998–2020 (N=27020)

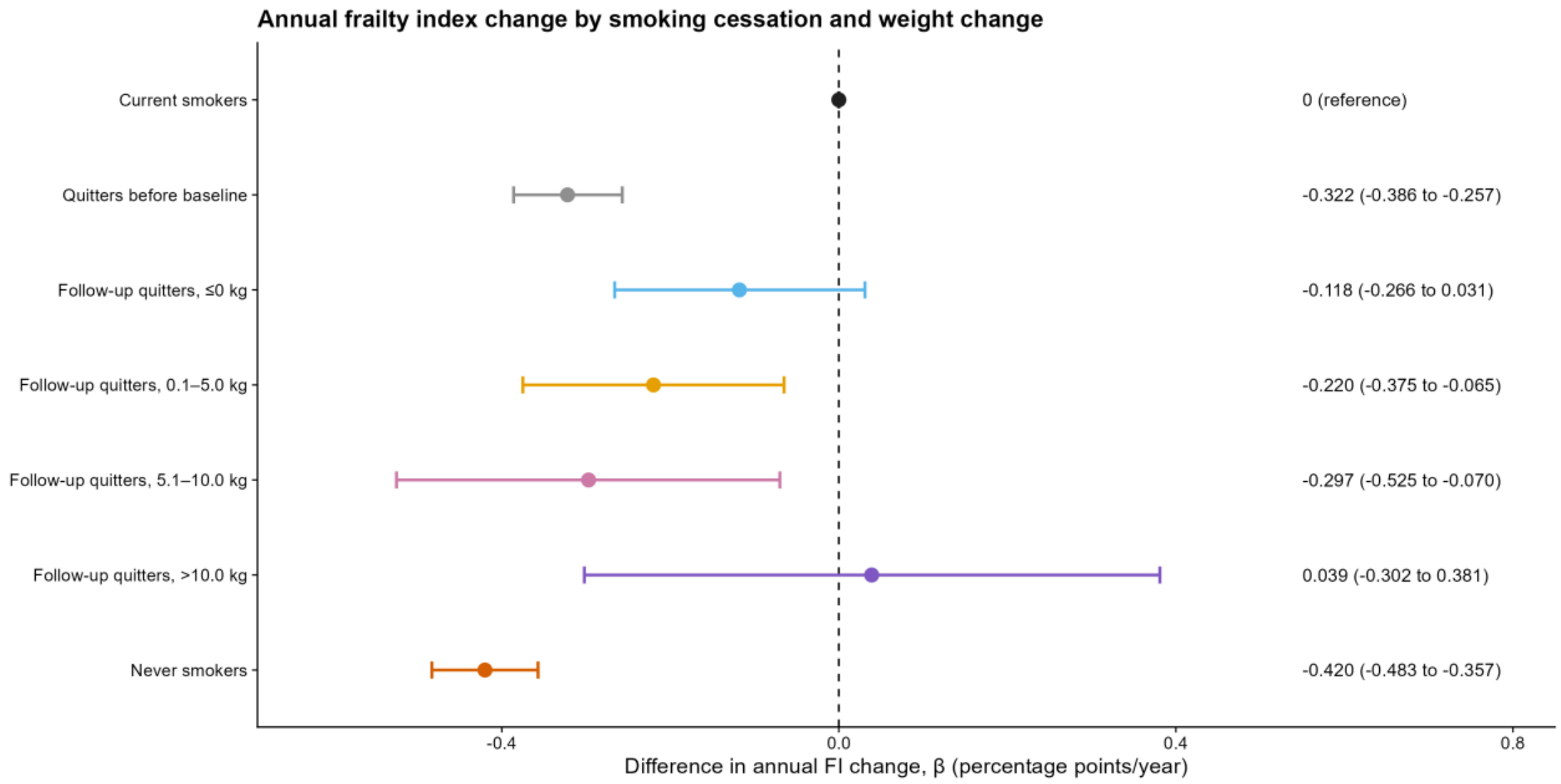

Points and horizontal lines show β coefficients and 95% confidence intervals from linear mixed-effects models for annualized frailty-index change, expressed as percentage points per year. Current smokers were the reference. Negative β values indicate slower annual frailty-index accumulation. CI, confidence interval; FI, frailty index; HRS, Health and Retirement Study.
